# Is there any association between Sarcoidosis and infectious agents?: a systematic review and meta-analysis

**DOI:** 10.1186/s12890-016-0332-z

**Published:** 2016-11-28

**Authors:** Tiago Esteves, Gloria Aparicio, Vicente Garcia-Patos

**Affiliations:** 1 Department of Medicine, Universitat Autònoma de Barcelona, Passeig de la Vall d’Hebron, 119-129, 08035 Barcelona, Spain; 2 Department of Dermatology, Hospital Universitari Vall d’Hebron, Barcelona, Spain

**Keywords:** Sarcoidosis, Propionibacterium acnes, Mycobacteria, Infection, Meta-analysis

## Abstract

**Background:**

During the last few years, investigators have debated the role that infectious agents may have in sarcoidosis pathogenesis. With the emergence of new molecular biology techniques, several studies have been conducted; therefore, we performed a meta-analysis in order to better explain this possible association.

**Methods:**

This review was conducted in accordance with the Preferred Reporting Items for Systematic Reviews and Meta-Analyses (PRISMA) statement from the Cochrane collaboration guidelines. Four different databases (Medline, Scopus, Web of Science, and Cochrane Collaboration) were searched for all original articles published from 1980 to 2015. The present meta-analysis included case–control studies that reported the presence of microorganisms in samples of patients with sarcoidosis using culture methods or molecular biology techniques. We used a random effects or a fixed-effect model to calculate the odds ratio (OR) and 95% confidence intervals (CI). Sensitivity and subgroup analyses were performed in order to explore the heterogeneity among studies.

**Results:**

Fifty-eight studies qualified for the purpose of this analysis. The present meta-analysis, the first, to our knowledge, in evaluation of all infectious agents proposed to be associated with sarcoidosis and involving more than 6000 patients in several countries, suggests an etiological link between *Propionibacterium acnes* and sarcoidosis, with an OR of 18.80 (95% CI 12.62, 28.01). We also found a significant association between sarcoidosis and mycobacteria, with an OR of 6.8 (95% CI 3.73, 12.39). *Borrelia* (OR 4.82; 95% CI 0.98, 23.81), HHV-8 (OR 1.47; 95% CI 0.02, 110.06) as well as *Rickettsia helvetica*, *Chlamydia pneumoniae*, Epstein-barr virus and Retrovirus, although suggested by previous investigations, were not associated with sarcoidosis.

**Conclusion:**

This meta-analysis suggests that some infectious agents can be associated with sarcoidosis. What seems clear is that more than one infectious agent might be implicated in the pathogenesis of sarcoidosis; probably the patient’s geographical location might dictate which microorganisms are more involved. Future investigations and more clinical trials are need to bring these evidences to a more global level.

**Electronic supplementary material:**

The online version of this article (doi:10.1186/s12890-016-0332-z) contains supplementary material, which is available to authorized users.

## Background

Sarcoidosis is a systemic disorder of unknown origin that is characterized by the presence of non-caseating granulomas. With worldwide distribution, more than one causative agent may be implicated in its pathogenesis [1], with numerous infectious and non-infectious etiological agents having been identified [2]. Currently, the focus is on infectious agents, especially species of *Mycobacterium* and *Propionibacterium*. Other infectious agents have been investigated with inconclusive or conflicting results, such as *Borrelia burgdorferi*, *Rickettsia helvetica, Chlamydia pneumoniae*, viruses, fungal infections, and *Leishmania* species [3–11].

There are only two relevant meta-analyses in the literature [12, 13], which address the causal relationship of some infectious agents in sarcoidosis. Since then, more than 20 new investigations have been published, thus adding new relevant data to the discussion. This meta-analysis is the first to evaluate all infectious agents that may be involved in sarcoidosis.

## Methods

### Search strategy

This review was conducted in accordance with the Preferred Reporting Items for Systematic Reviews and Meta-Analyses (PRISMA) statement from the Cochrane collaboration guidelines. A checklist is available (Additional file 1). Since this study was a literature review and meta-analysis of previously reported studies, ethical approval or additional consent from participants was not required. Four different databases (Medline, Scopus, Web of Science and Cochrane Database) were searched for all original articles without language restriction published from January 1980 to May 2015, using the search strategy described in online supplementary data (Additional file 2).

### Inclusion criteria

The inclusion criteria were as follows: (i) the diagnosis of sarcoidosis was made according to the classical criteria: a compatible clinical and radiological picture, histopathological demonstration of non-caseating granulomas with negative stains for mycobacterium and fungi, and exclusion of other granulomatous diseases; [14] (ii) case–control studies that reported the presence of microorganisms in samples, both histological and cellular, of patients with sarcoidosis, using either culture methods (direct isolation of the organism) or molecular biology techniques (analysis of DNA, RNA or proteins); (iii) odds ratios (OR) and the corresponding confidence intervals (CI) or sufficient information to calculate them; (iv) patients without sarcoidosis were used as a reference group.

### Exclusion criteria

Studies involving other techniques (e.g. ELISA, immunohistochemistry and immunofluorescence) were excluded from the analysis.

### Data extraction

First, two independent authors (T. Esteves and V. Garcia-Patos) reviewed all titles and abstracts. A second selection was based on a full-text review of potentially relevant articles and any disagreement was resolved by discussion between the three authors of this meta-analysis. A standardized data collection form was used to extract the following items: author(s), title of article, study design, year of publication, country of origin, study size, details of molecular or other techniques used.

### Statistical analysis and methodological quality assessment

The measure of interest was the OR and 95% CI calculated from each study, in order to assess the presence of microorganisms in sarcoidosis samples versus controls. Data analyses were performed using Stata Statistical Software 2015 (StataCorp LP, College Station, Texas, USA). We used a random-effects model to calculate the OR and 95% CI from each study [15].

We assessed the heterogeneity among studies using Cochran’s Q test [16], complemented by the I^2^-test. [17] An I^2^ value of 76–100% represents high heterogeneity, 51–75% moderate heterogeneity and 0–50% low or insignificant heterogeneity [17]. If the result of the Chi-square heterogeneity test was not significant (*p* > 0.10), we used the fixed-effects model described by Mantel and Haenszel [18] to calculate the pooled OR estimate. Additionally, sensitivity and subgroup analyses were performed in order to explore the heterogeneity among studies.

## Results

### Studies included

A total of 2465 articles were identified from the initial electronic search using the outlined search term parameters (Additional file 2). Among these, 2401 studies were excluded because they did not meet the inclusion criteria. A total of 64 articles were identified as investigating the role of infectious agents in sarcoidosis using either microbial culture or molecular methods. Six of these were later excluded since they were descriptive studies without a control group. Therefore, 58 case–control studies were qualified for the analysis according to the inclusion and exclusion criteria. Additional file 3 summarizes the study flow.

In total, the 58 studies involved 2467 samples from patients with proven sarcoidosis and 3656 samples from control patients with other non-sarcoid disorders. All studies used molecular techniques to identify the different types of infectious agents except for two, which used microbial culture in their analyses [19, 20].

With regard to the infectious agents investigated, 36 studies evaluated the presence of mycobacteria [20–55] (Table 1), 11 evaluated *P. acnes* [19, 22, 24, 25, 31, 35, 38, 56–59] (Table 2), seven evaluated human herpesvirus-8 (HHV-8) [22, 40, 60–64] (Table 3), and six evaluated *Borrelia* species [4, 65–69] (Table 4). Other infectious agents were investigated in some of the studies included, but there were insufficient cases to perform a meta-analysis. Three studies evaluated the presence of *Rickettsia* species, and one found a strong association between *Rickettsia helvetica* and sarcoidosis [70] (OR 21.72; CI:1.23–384.74). The second study did not reveal a significant association [3] (OR 0.43; CI:0–23.23), while in the third, all real-time PCR analyses for the detection of Rickettsia were negative [71]. None of the studies reported a significant association with *Chlamydia pneumonia* [7, 8, 72], Epstein-Barr virus [40], or retrovirus [73].

**Table 1:** Case–control studies evaluating the role of mycobacteria in sarcoidosis

First author/Year (Ref.)	Country	Molecular technique	Sarcoidosis patients		Non-sarcoidosis controls		OR (95% CI)
			n/N	Type of microorganisms	n/N	Type of microorganisms	
Bocart, 1992 [23]	France	PCR of 65 kDa mycobacterial antigen and IS6110	2/22	MTBC	0/22	-	5.49 (0.25–121.18)
Hofland, 2014 [20]	Netherlands	NAAT for Mycobacteria and Culture	0/32	-	2/86	1 MTBC, 1NTM	0.52 (0.02–11.13)
Robinson, 2013 [24]	USA	PCR for 16S rDNA, hsp65 and rpoB	2/30	NTM	1/30	NTM	2.07 (0.18–24.15)
Oswald-Richter, 2012 [25]	USA	MALDI-IMS for ESAT-6	5/15	Mycobacterium spp	0/4	-	4.71 (0.21–104.49)
Svendsen, 2011 [26]	Denmark	BD ProbeTec IS6110 amplification	1/52	MTBC	0/50	-	2.94 (0.12–73.93)
Mootha, 2010 [27]	India	PCR of 65 kDa mycobacterial antigen and IS6110	13/27	10 MTBC, 3 NTM	2/40	NTM	17.64 (3.53–88.25)
Zhou, 2008 [28]	China	Real-time PCR of IS986 and human β-blobin gene	20/104	MTBC	7/55	MTBC	1.63 (0.64–4.14)
Dubaniewicz, 2006 [29]	Poland	BD ProbeTec IS6110 amplification	3/50	MTBC	0/10	-	1.55 (0.07–32.27)
Fite, 2006 [30]	Spain	PCR of IS6110 and Southern blot hybridisation	9/23	MTBC	1/23	MTBC	14.14 (1.61–124.11)
Yasuhara, 2005 [31]	Japan	PCR of IS6110	0/6	-	0/6	-	-
Song, 2005 [32]	USA	PCR of MTB 16S rRNA	6/16	MTBC	0/16	-	20.43 (1.04–401.67)
Marcoval, 2005 [33]	Spain	NAAT for rRNA of MTBC	0/35	-	0/39	_	-
Yu-Yun Lee, 2002 [34]	Taiwan	Nested PCR for mycobacterial hsp65 DNA	7/21	NTM	0/16	-	17.07 (0.89–325.59)
Drake, 2002 [21]	USA	PCR of 16S rRNA, rpoB and IS6110	15/25	11 MTBC, 3 NTM, 1 both	0/25	_	75.29 (4.12–1377.06)
Gazouli, 2002 [22]	Greece	PCR of IS6110/IS1245/IS900/IS901, 16S rRNA, MPB64 and mtp40	33/46	MTBC	0/20	-	101.74 (5.74–1804.62)
Eish, 2002 [35]	Japan	PCR of IS6110/IS900	5/108	MTBC	2/86	MTBC	2.04 (0.39–10.78)
Klemen, 2000 [36]	Austria	PCR of IS6110 and mycobacterial chaperonin	3/4	NTM	0/39	_	184.33 (6.26–5425.48)
Li, 1999 [37]	USA	PCR of 65 kDa mycobacterialantigen and RFLP analysis	16/20	2 MTBC, 14 NTM	0/20	_	150.33 (7.54–2997.83)
Ishige, 1999 [38]	Japan	PCR of IS6110	3/15	MTBC	1/15	MTBC	3.50 (0.32–38.23)
Wilsher, 1998 [39]	NZ	PCR of IS6110, nested PCR to amplify 85 bp sequence within the 123 bp product	0/31	_	0/10	-	-
Di Alberti, 1997 [40]	Italy	Heminested PCR for 16S rRNA	17/38	4 NTM, 13 Mycobacterium spp	39/113	39 Mycobacterium spp	1.54 (0.73–3.24)
Vokurka, 1997 [41]	France	PCR of IS6110 and DR region	0/15	_	0/27	_	-
Ozcelik, 1997 [42]	Turkey	PCR of IS6110	5/11	MTBC	2/15	MTBC	5.42 (0.81-36.36)
Popper, 1997 [43]	Austria	PCR of 65 kDa mycobacterial antigen and IS6110	11/35	NTM	0/39	-	37.08 (2.09–657.90)
El-Zaatari, 1996 [44]	USA	PCR of IS900/IS902, MAC-specific PCR assay and Western blot	7/7	NTM	13/38	NTM	28.33 (1.50–534.74)
Fidler, 1993 [45]	UK	PCR of 65 kDa mycobacterial antigen and IS6110	7/16	MTBC	1/16	MTBC	11.67 (1.23–110.95)
Thakker, 1992 [46]	UK	PCR of groEL	1/14	MTBC	1/11	MTBC	0.77 (0.04–13.87)
Gerdes, 1992 [47]	Germany	PCR of 16S rDNA	0/14	-	0/10	-	-
Mitchell, 1992 [48]	UK	Mycobacterial rRNA detection by liquid phase hybridisation	5/5	MTBC	0/5	-	121 (2.02–7259.18)
Saboor, 1992 [49]	UK	PCR of IS986/IS6110 and groEL	14/20	10 MTBC, 4 NTM	5/22	3 MTBC, 2 NTM	7.93 (1.99–31.59)
Lisby, 1993 [50]	Denmark	Nested PCR for IS900	0/18	-	0/18	-	-
Grosser, 1999 [51]	Germany	PCR of IS986/IS6110	35/65	MTBC	1/34	MTBC	38.50 (4.96–298.57)
Vago, 1998 [52]	Italy	PCR of IS6110	2/30	MTBC	0/17	-	3.07 (0.14–67.75)
Richter, 1996 [53]	Germany	PCR of mycobacterial 16S rDNA	1/24	MTBC	3/57	MTBC	0.78 (0.08–7.93)
Ghossein, 1994 [54]	USA	PCR of 65 kDa mycobacterial antigen	0/10	-	0/10	-	-
Cannone, 1997 [55]	Italy	PCR of IS6110	2/30	MTBC	0/10	-	1.84 (0.08–41.62)

*n* Mycobacteria-positive samples, *N* total samples, *PCR* polymerase chain reaction, *65 kDa* 65-Kilodalton mycobacteria antigen, *IS6110* insertion sequence to identify *Mycobacterium tuberculosis* complex (MTBC), *NTM* non-tuberculous mycobacteria, *NAAT* nucleic acid amplification test, *16S rDNA* ribosomal DNA common to all mycobacteria, *rpoB* RNA polymerase β-subunit gene, *MALDI-IMS* matrix-assisted laser desorption ionization as a mass spectrometry imaging, *ESAT-6* 6 kDa early secretory antigenic target produced by Mycobacterium tuberculosis, *IS986* insertion sequence to identify MTBC, *rRNA* ribosomal RNA, *IS1245/IS900/IS901/IS902* insertion sequence to identify *Mycobacterium avium* complex, *MPB64* mycobacterial protein, *mtp40* Specific primers of MTB species, *RFLP* restriction fragment length polymorphism *DR* direct repeat, *groEL* gene encoding 65 kDa antigen

**Table 2:** Case–control studies evaluating the role of *P. acnes* in sarcoidosis

First author/Year (Ref.)	Country	Molecular technique	Sarcoidosis	Controls	OR (95% CI)
			n/N	n/N	
Robinson, 2013 [24]	USA	PCR for bacterial 16S rDNA	7/30	1/30	8.83 (1.01–76.96)
Oswald-Richter, 2012 [25]	USA	MALDI-IMS for propionibacterial proteins	7/15	1/4	2.63 (0.22–31.35)
Yasuhara, 2005 [31]	Japan	PCR for 16S rRNA	2/6	0/6	7.22 (0.28–189.19)
Gazouli, 2002 [22]	Greece	PCR for 16S rRNA	0/46	0/20	-
Eish, 2002 [35]	Japan	PCR for 16S rRNA	93/108	25/86	15.13 (7.39–30.99)
Ishige, 1999 [38]	Japan	Quantitative PCR for 16S rRNA	12/15	3/15	16 (2.67–95.75)
Negi, 2012 [56]	Japan	Immunohistochemical methods (PAG and TIG antibodies) and western blot	149/196	0/79	500.43 (30.44–8226.20)
Yamada, 2002 [57]	Japan	Quantitative real-time PCR for 16S rRNA	8/9	2/9	28 (2.07–379.25)
Eishi, 1994 [58]	Japan	PCR for P. acnes DNA	36/39	12/29	17 (4.23–68.28)
Abe, 1984 [19]	Japan	Isolation of P acnes in culture	31/40	38/180	12.87 (5.65–29.34)
Hiramatsu, 2003 [59]	Japan	Nested PCR for 16S rRNA	21/30	7/30	7.67 (2.42–24.24)

*16S rDNA* ribosomal DNA, *MALDI-IMS* matrix-assisted laser desorption ionization as a mass spectrometry imaging, *rRNA* ribosomal RNA

**Table 3:** Selected studies on the association between HHV-8 and sarcoidosis

First author/Year (Ref.)	Country	Molecular technique	Patients	Controls	OR (95% CI)
			n/N	n/N	
Knoell, 2005 [60]	USA	PCR for HHV-8 DNA	0/8	0/8	-
Gazouli, 2002 [22]	Greece	PCR for HHV-8 DNA	0/46	0/20	-
Fredricks, 2002 [61]	USA	PCR for HHV-8 ORF 26 DNA	0/18	0/4	-
Maeda, 2000 [62]	Japan	Hemi-nested PCR for HHV-8 DNA	4/119	4/120	1.01 (0.25–4.13)
Sugaya, 1999 [63]	Japan	Nested PCR for HHV-8 ORF 26 DNA	0/12	1/1	0.01 (0.00–0.95)
Bélec, 1998 [64]	France	Nested PCR for HHV-8 ORF 25/26 DNA	0/14	2/17	0.21 (0.01–4.84)
Di Alberti, 1997 [40]	Italy	Nested PCR for HHV-8 ORF 26 DNA and Heminested PCR for HHV-8 ORF 25 DNA	38/39	6/113	677.67 (79.01–5812.52)

*HHV-8* Human Herpesvirus 8, *ORF 25/26 DNA* insertion sequence to identify HHV-8

**Table 4:** Selected studies on the association between *Borrelia* species and sarcoidosis

First author/Year (Ref.)	Country	Molecular Technique	Sarcoidosis		Controls		OR (95% CI)
			n/N	Type of microorganism	n/N	Type of microorganism	
Derler, 2009 [4]	Austria	Focus-floating microscopy and Borrelia-specific PCR DNA	13/35	Borrelia sp.	1/61	Borrelia sp.	35.45 (4.38–287.16)
Ishihara, 1998 [65]	Japan	Dot-blot analysis (Dotblot Borrelia Kit)	15/46	Borrelia sp.	2/100	Borrelia sp.	23.71 (5.14–109.46)
Martens, 1997 [66]	Germany	Western blot for Borrelia burgdorferi	1/60	Borrelia burgdorferi	27/1000	Borrelia burdorferi	0.61 (0.08–4.57)
Lian, 1995 [67]	China	PCR for Borrelia burgdorferi DNA	6/49	Borrelia burgdorferi	2/28	Borrelia burgdorferi	1.81 (0.34–9.66)
Xu, 1996 [68]	China	In situ PCR for Borrelia burgdorferi DNA	0/23	-	0/23	-	-
Ishihara, 1996 [69]	Japan	Elisa and Dot-blot analysis for Borrelia sp.	1/38	Borrelia sp.	1/80	Borrelia sp.	2.14 (0.13–35.08)

### Meta-analysis

#### Mycobacteria (Table 1)

Both *Mycobacterium tuberculosis* complex (MTBC) and nontuberculous mycobacteria (NTM) were investigated in most of the 36 relevant studies, although some used primers to detect only *M. tuberculosis* [26, 28–33, 38, 39, 41, 42, 51, 52, 55], and others detected only nontuberculous mycobacteria [44, 50].

**Fig. 1:**
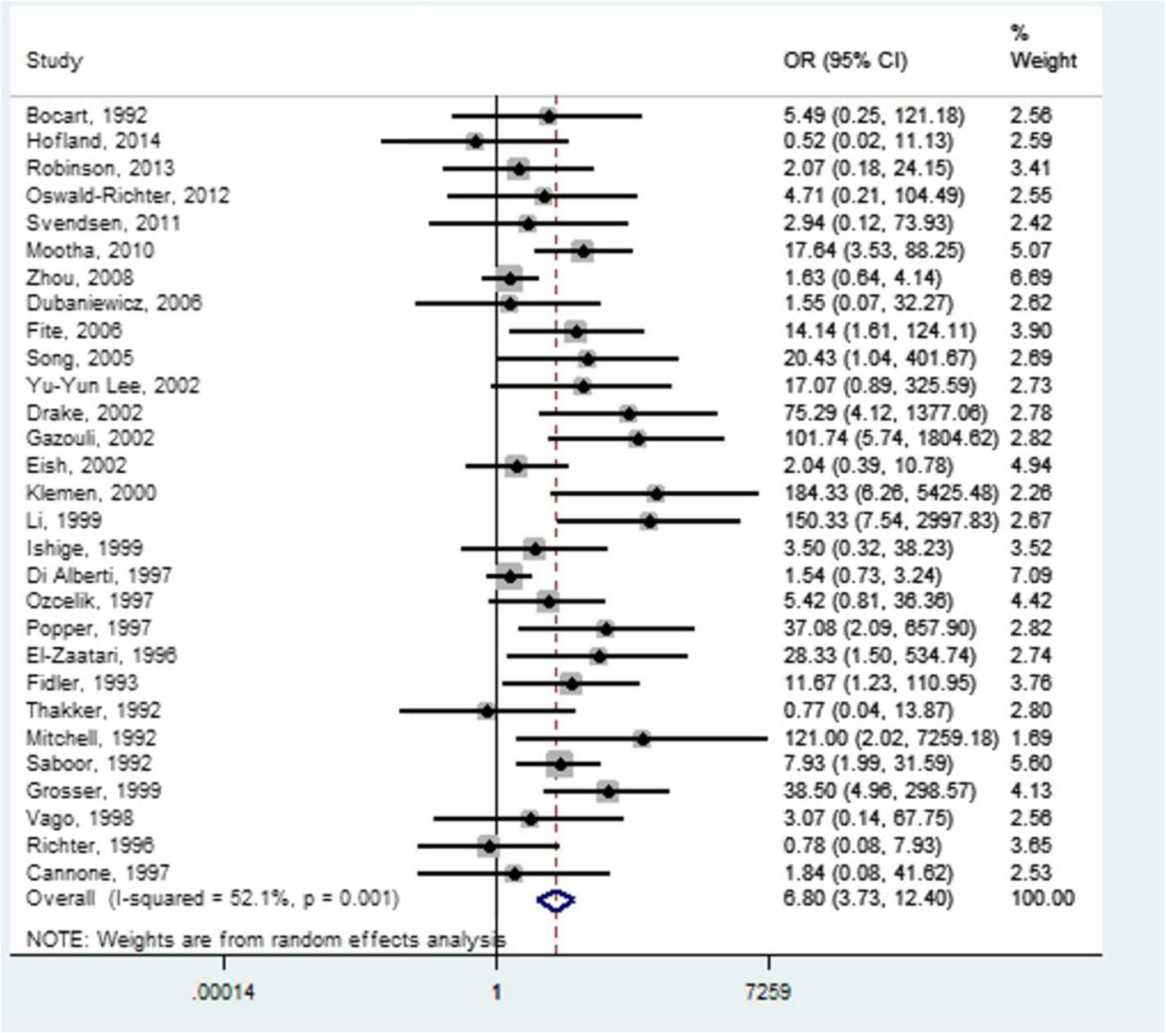
Forest plot of studies that show the presence of mycobacteria in sarcoidosis patients versus controls

Figure 1 provides a forest plot for sarcoidosis and mycobacteria based on a total of 1034 sarcoidosis patients and 1054 controls. Of the 1034 sarcoidosis cases, 173 were positive for MTBC, and 58 were positive for NTM. It was not possible to identify the type of mycobacteria involved in 18 samples, while both types of mycobacteria DNA were present in one sample. In total, 250 sarcoidosis samples were positive for some form of mycobacteria DNA sequence for a positive signal rate of 24.2%. We found a significant association between sarcoidosis and mycobacteria with an OR of 6.8 (95% CI:3.73–12.39). A strong association was also found between sarcoidosis and NTM alone with an OR of 10.39 (95% CI:5.25–20.56), as well as for *M. tuberculosis* complex (OR 4.29; CI:2.60–7.08). There was moderate heterogeneity among studies (I^2^ test 52.1%; *p* = 0.001), although all but three studies estimated a risk above unity with significance in most cases.

#### *P. acnes* (Table 2)

**Fig. 2:**
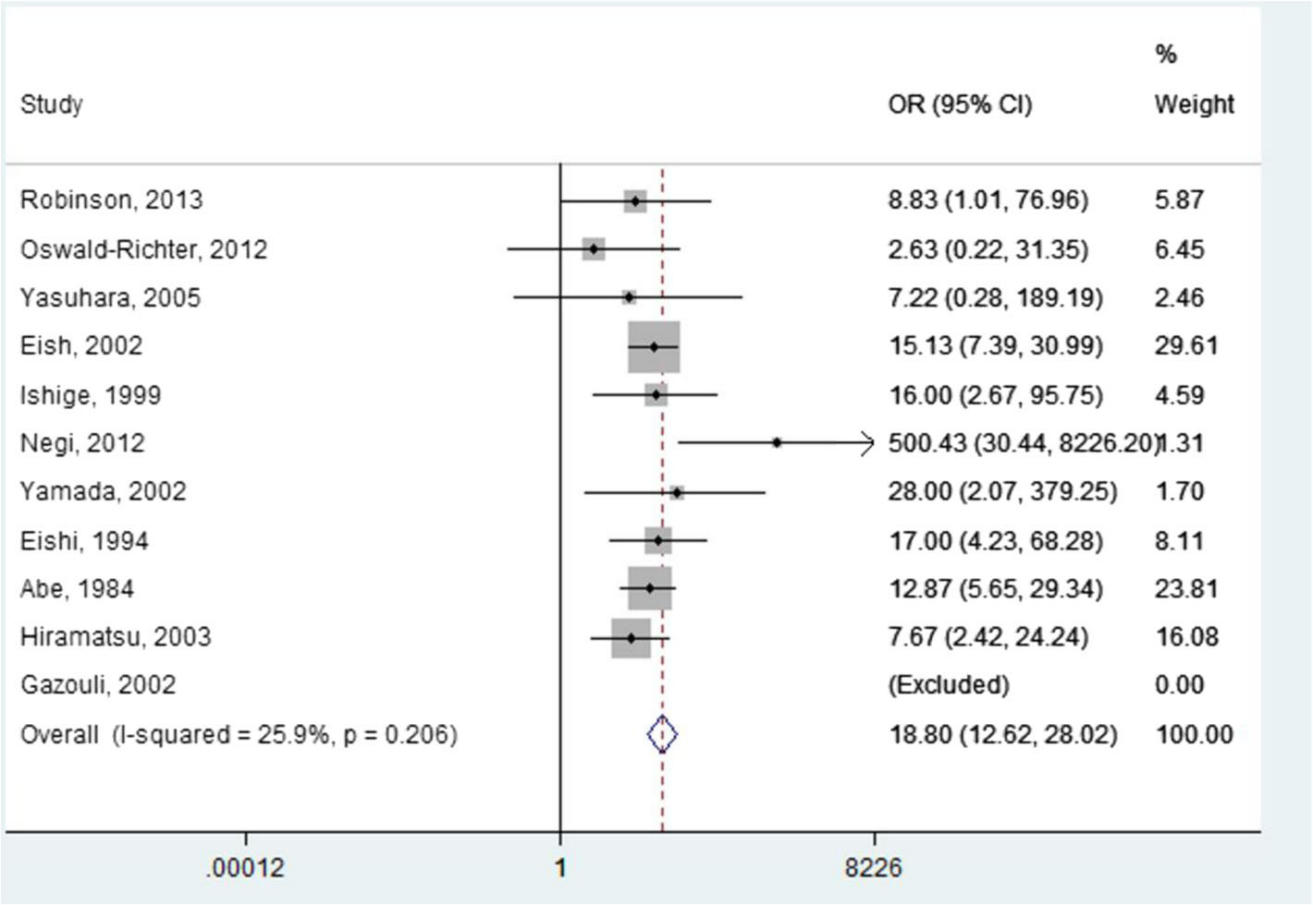
Forest plot of studies that show the presence of *P. acnes* in sarcoidosis patients versus controls

The risk of sarcoidosis associated with *P. acnes* was provided by the study design (Fig. 2). The OR derived from 11 studies with 534 cases and 488 controls was 18.80 (95% CI:12.62–28.01), and there was low heterogeneity (I^2^ test 25.9%; *p* = 0.206). There was a positive signal rate of 68.54% for *P. acnes* (366 positive samples from 534 patients). When accounting for the source of biological samples studied, we found that nine of the 11 studies [19, 22, 24, 25, 35, 38, 56–58] evaluated the presence of *P. acnes* in lymph node samples, of which seven evaluated this location exclusively [19, 24, 35, 38, 57, 58]. This could justify the low heterogeneity among studies, contrary to what was observed in the forest plot of mycobacteria, where the studied biological samples were more heterogeneous.

#### *Borrelia* and HHV-8 (Tables 3 and 4)

**Fig. 3:**
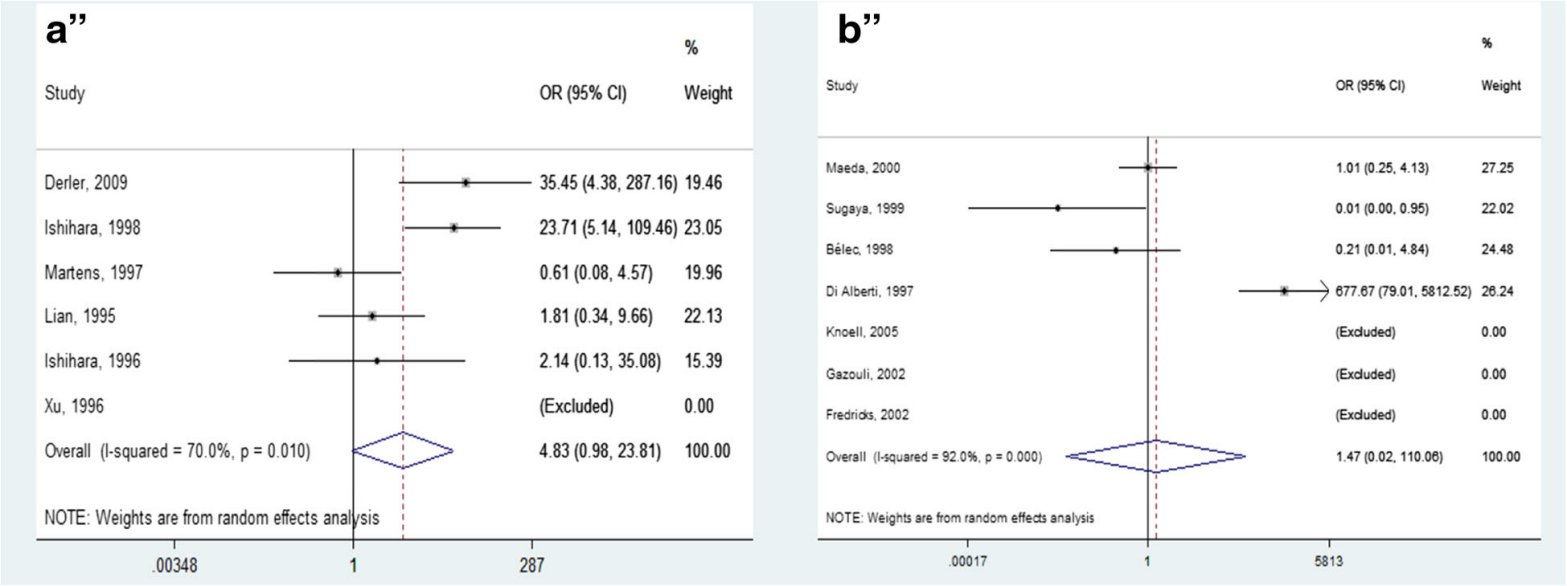
Forest plot: **a** summary OR for the presence of *Borrelia* species in sarcoidosis patients; **b** summary OR for the presence of HHV-8 in sarcoidosis patients

Of the six articles assessing the presence of *Borrelia* in sarcoidosis tissues, three used polymerase chain reaction (PCR) techniques for DNA amplification of *B. burgdorferi* [66–68], whereas the other three did not specify which species of *Borrelia* were involved [4, 65, 69]. The pooled OR derived from these six studies with 251 cases and 1292 controls was 4.82, but this result did not reach statistical significance (95% CI:0.98–23.81). Statistical heterogeneity was moderate with an I^2^ of 70% and *p* = 0.01 Fig. 3 a).

Di Alberti et al [40] were the only ones to report a significant association between sarcoidosis and HHV-8 in comparison with controls. However, the remaining six studies refuted those results [22, 60–64]. Overall, there was no significant association between sarcoidosis and HHV-8 (OR 1.47; CI:0.02–110.06), and there was high heterogeneity among studies (I^2^ test 92%; *p* = 0.000) (Fig. 3 b).

### Evaluation of publication bias

**Fig. 4:**
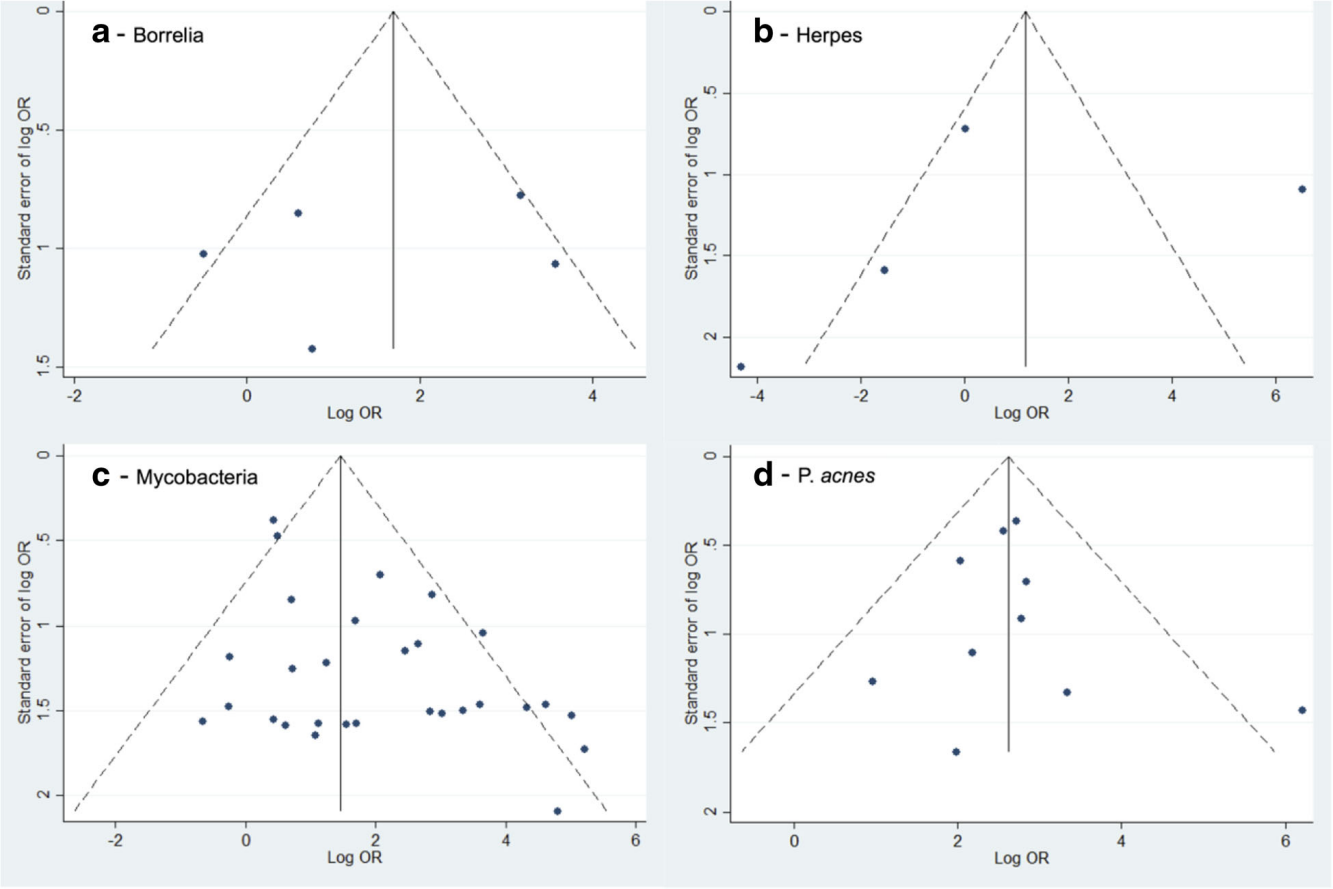
Funnel plot of all studies: (a) Borrelia; (b) Herpes; (c) Mycobacteria; (d) P. acnes

We performed funnel plots to evaluate publication bias (Fig. 4). The funnel plots of HHV-8 and mycobacteria showed evidence of publication bias (Fig. 4b and c), while the graphs regarding the presence of *Borrelia* and *P. acnes* are fairly symmetrical (Fig. 4a and d). Thus, no suggestion of publication bias is indicated in these cases.

### Sensitivity and subgroup analysis

To verify the robustness of the results, as well as the potential sources of heterogeneity, subgroup and sensitivity analyses were performed (especially for mycobacteria).

#### Subgroup analysis

Concerning the studies of mycobacteria, we conducted subgroup meta-analysis by various study characteristics (Table 5). The pooled OR was calculated in subgroups of studies according to geographical area, publication year, type of study, and molecular technique used. There was a significant association between sarcoidosis and mycobacteria in all subgroups, except in three studies included in the subgroup of molecular techniques (BD ProbeTec and culture). The pooled OR was significantly higher with some covariates, however almost all of the ORs derived from these subgroup data were significantly above unity.

**Table 5:** Subgroup and sensitivity analysis of the association between sarcoidosis and mycobacteria

	No. of studies	OR (95%CI)	*P-*value heterogeneity	*I* ^2^ (%)
Subgroup analysis				
1 - Geographical region				
Europe	22	6.92 (3.05, 15.71)	0.004	53.8
USA	7	18.21 (4.64, 71.53)	0.238	26.2
Asia	7	4.09 (1.38, 12.12)	0.093	49.8
2 - Publication year				
< 2000	20	6.63 (2.84, 15.51)	0.006	54.2
> =2000	16	8.40 (3.31, 21.31)	0.012	53
3 – Type of study				
Prospective	10	11.91 (4.94, 28.69)	0.743	0.0
Retrospective	26	6.41 (3.14, 13.09)	0.001	57.1
4 - Molecular technique				
PCR	29	7.04 (3.57, 13.89)	0.000	58.8
Hybridization	2	37.81 (3.40, 420.43)	0.484	0.0
Protein analysis	2	12.12 (1.44, 102.20)	0.408	0.0
BD ProbeTec	2	2.09 (0.23, 19.10)	0.776	0.0
Culture	1	0.52 (0.02, 11.13)	-	-
Sensitivity analysis				
1 - Biological samples				
Only lymph nodes	11	3.82 (1.53, 9.49)	0.384	4.0
Only lung	5	2.93 (1.09, 7.86)	0.098	56.9
Only skin	2	11.58 (0.06, 2016.91)	0.021	81.3
2 – Incidence of tuberculosis				
Only countries with low burden of TB	33	4.33 (2.06, 9.10)	0.042	45.7

*CI* confidence interval, *OR* odds ratio, *BD ProbeTec* molecular detection based on strand displacement amplification (SDA) technology, *TB* tuberculosis

Through the subgroup analyses, it was noted that the variables that most influenced the results of heterogeneity were: a) the study type, being null the heterogeneity in the ten prospective studies, contrasting with the moderate heterogeneity in retrospective studies; b) the geographical location, verifying a low heterogeneity in studies conducted in the USA and Asia.

#### Sensitivity analysis

We also performed a sensitivity analysis to complement the subgroup analysis in order to better explain the heterogeneity between studies (Table 5).

Regarding sarcoidosis and mycobacteria, there was a strong and significant association (OR 4.33; CI:2.06–9.10) in subgroup analysis of geographic locations when we restricted to studies performed in countries with low burden of tuberculosis [24, 30, 35, 38, 40, 42, 44–46, 49, 51, 53]. There was low heterogeneity among these studies (I^2^ test 45.7%; *p* = 0.042) (Table 5).

Another subgroup analysis compared the results according to the different biological samples used. Most of the studies included in our meta-analysis used biological samples from different locations, including the skin, lymph nodes, and lungs. However, when we restricted the analysis to include only studies that performed PCR on biological samples of the same type, the associations were also significant in both lung samples [30, 40, 45] (OR 2.93; CI:1.09–7.86; I^2^ = 56.9%) and lymph node samples [24, 35, 38, 40, 46] (OR 3.82; CI:1.53–9.49; I^2^ = 4%), but not skin biological specimens [37, 40] (OR 11.58; CI:0.06–2016.91; I^2^ = 81.3%). Once again, the heterogeneity among studies was low to moderate except in two studies performed on skin biopsies (Table 5).

In sensitivity analysis on studies of sarcoidosis and *P. acnes*, we found a significant association compared with the controls when we selected only studies performed outside Asia [22, 24, 25], with a pooled OR of 5.5 (95% CI:1.13–27.42) and no heterogeneity among studies.

## Discussion

The present meta-analysis is the first to evaluate all infectious agents proposed to be associated with sarcoidosis and involving more than 6000 patients in several countries. The results point to an etiological link between *P. acnes* and sarcoidosis with a positive signal rate of 68.54%. Also, almost one quarter of sarcoidosis patients show the presence of mycobacteria within the lesions. The associations are fairly specific, since *P. acnes* (OR 18.80) and mycobacteria (OR 6.8) were significantly increased in sarcoidosis patients, while *Borrelia* (OR 4.82; CI:0.98–23.81) and HHV-8 (OR 1.47; CI:0.02–110.06) were not associated with sarcoidosis, contrary to previous investigations.

Three decades ago, Abe et al [19] reported that *P. acnes* was the only bacterium isolated in lymph node biopsy samples taken from sarcoidosis patients. Studies published in recent years have confirmed that *P. acnes* could be a possible infectious agent implicated in the pathogenesis of sarcoidosis [24, 56, 74–76]. However, some studies suggest that *P. acnes* is not specific for sarcoidosis because it is a normal inhabitant of peripheral lung tissue and mediastinal lymph nodes, apart from the skin [77]. Despite this, the results of our meta-analysis show a significant quantitative difference in the presence of the *P. acnes* genome in sarcoidosis patients compared to control subjects. This suggests that this microorganism may be present abnormally or may proliferate ectopically in such sarcoid lesions.

However, it is important to note that most of the studies in our meta-analysis evaluating the role of *P. acnes* in sarcoidosis were by Japanese groups testing Japanese patients, while only very limited data exist for African American or Caucasian patients [22, 24, 25]. The results were conflicting in these three studies, but interestingly, the pooled OR was above unity and statistically significant (5.58; CI:1.13–27.42). Despite these surprising results, the ORs observed in studies with Japanese patients were far superior, and the results were more consistent and robust. Differences between these two groups may be due to the geographical, ethnic, or racial composition of the study population. Sarcoidosis in Japanese patients is characterized by a high rate of ocular, cutaneous, and cardiac involvement, while in Europe and the USA, this disease mainly affects the lungs.

In 2002, the first large, relevant study was published as a collaboration between several countries [35]. The results of this international study suggest an association between *P. acnes* and sarcoidosis in not only Japanese patients (positive signal rate of 89.2%), but also in Europeans (positive signal rate of 81.4%). However, more international corporative studies with quantitative PCR are needed to clarify the role of *P. acnes* in sarcoidosis and for better understanding of the phenotypic variability of this disease.

Recent years have witnessed substantial discussion among investigators about the role that mycobacteria may have in the pathogenesis of sarcoidosis, and the issue remains unsettled, if not controversial. With the emergence of new microbiological techniques, especially in the molecular biology area, several studies have been conducted in order to investigate this possible association more deeply.

In the present meta-analysis, we identified 36 studies assessing the presence of mycobacteria in a total of 1034 sarcoidosis patients and 1054 controls. The results suggest a strong association of sarcoidosis with NTM (OR 10.39; CI:5.25–20.56) and with MTBC (4.29; CI:2.60–7.08). However, to evaluate the possible relationship between mycobacteria and sarcoidosis, the current incidence of tuberculosis should be taken into account in general populations of the different countries where the studies of sarcoidosis were performed. In the sensitivity analyses, a significant association was also found (OR 4.33; CI:2.06–9.10) when we restricted the analysis to include only studies performed in countries with low prevalence of tuberculosis. This further confirms the robustness of the results and the relevance of this association worldwide.

Despite the heterogeneity of analyzed studies and the potential publication bias suggested by the mycobacteria funnel plots, most of the ORs derived from individual data were significantly above unity. Furthermore, sensitivity and subgroup analyses including only studies performed on lung samples or lymph nodes showed low heterogeneity. Therefore, it is important to account for the heterogeneity in sarcoidosis specimens (lung versus skin or lymph nodes). We found significant increased ORs in studies performed on lung or lymph node samples but not in skin specimens. Possible explanations for this include the following: 1) In the initial phase of the disease, systemic sarcoidosis primarily affects and spreads through the lymphatic system, following the lymphatic vessels to the hilar and mediastinal lymph nodes. 2) Lung and lymph node samples are obtained sterilely by endoscopy biopsies and thus avoid possible microorganism contamination, in contrast to skin biopsies. 3) The two studies performed on sarcoidosis skin samples were both retrospective [37, 40]. In such studies, there is a greater possibility of both contamination of the paraffin-embedded specimens and more DNA fragmentation. In contrast, several studies performed on lung and lymph node samples were prospective, and only fresh tissues were used. Additionally, when we conducted the subgroup analysis according to the type of study, it was found a low heterogeneity in the 10 prospective studies (I^2^ = 0%), contrasting with the moderate heterogeneity in retrospective studies (I^2^ = 57.1%).

The hypothesis that *B. burgdorferi* could be a possible causal infectious agent for sarcoidosis was first mentioned in 1989 in epidemiological studies [78]. Since then, several studies have been conducted using serological or molecular techniques in order to clarify the role of *Borrelia* in the pathogenesis of sarcoidosis. We identified six articles assessing the presence of *Borrelia* in sarcoidosis tissues using molecular techniques (251 cases and 1292 controls), and we did not find a significant association (OR 4.82; CI:0.98–23.81). On the other hand, the two studies that reported a significant association between *Borrelia* and sarcoidosis [4, 65] were both conducted in regions where Lyme disease is endemic, in contrast to the four other articles performed in non-endemic areas [66–69], where the results did not reach statistical significance.

It is important to note that the frequency of exposure to *Borrelia. spirochete* is different between patients living in regions where the disease is endemic and those in regions where it is not. Thus, in countries with elevated *B. burgdorferi* prevalence, a protective immunity against this microorganism has to be assumed in the general population. T-helper lymphocyte activity to this microorganism might be a trigger for the development of sarcoidosis in endemic regions, which could explain the positive results in studies published in Austria and Japan [4, 65]. Apart from these two studies, the fact that significant positive PCR results could not be found argues against the hypothesis of a connection between *B. burgdorferi* infection and sarcoidosis. However, more studies are needed to clarify the possible association, especially in endemic areas.

There are several clinical implications of this study. Currently, immune suppression remains the primary treatment modality for sarcoidosis. Given our meta-analysis, it is worth exploring whether certain antibacterial or antimycobacterial drugs might alter the course of sarcoidosis. In the past, some clinical trials have been published with conflicting results using classical antituberculous drugs, such as isoniazid, amino-salicylic acid, and streptomycin [79–82]. Recently, Drake et al [83] conducted a double-blind, placebo-controlled study to investigate the efficacy of oral antimycobacterial therapy (levofloxacin, ethambutol, azithromycin, and rifampin) in patients with cutaneous sarcoidosis. The results were promising, with significant reductions in cutaneous lesion size. The same authors also conducted an open-label investigation using the same therapy regimen in pulmonary sarcoidosis patients, and the results were again very interesting with significant improvements in forced vital capacity from baseline to completion of therapy [84].

Other antimicrobial agents such as minocycline and doxycycline have been shown to be quite effective in treating cutaneous sarcoidosis in some series [85, 86]. However, the exact mechanism of action of these drugs it is not fully understood [87].

Currently, other clinical trials are being done (NCT02024555 and NCT01245036) to clarify the role that antimicrobial agents might have in the treatment of sarcoidosis.

Several limitations in our study should be recognized. First, one of the main potential limitations relates to the variability and heterogeneity of the results analyzed. It is important to consider that the majority of these studies were assessed retrospectively and that data were obtained from different databases and hospitals. This could lead to different types of bias in the included studies and to variability in the results. Second, the risk of contamination or DNA fragmentation in PCR techniques can lead to false positive or false negative results. In addition, PCR does not discriminate between living and dead microorganisms. Third, the patients had varied clinical manifestations of sarcoidosis; moreover, the non-sarcoidosis controls were comprised of different types of subjects across the studies, which may cause misclassification bias and heterogeneity.

## Conclusion

The present meta-analysis, involving more than 6000 patients from various countries worldwide, suggests a significant association between sarcoidosis and some infectious agents, taking into account the marked difference in the percentage of microbial DNA-positive samples in sarcoidosis patients versus controls, especially mycobacteria (OR 6.8) and *P. acnes* (OR 18.80). Furthermore, our study also suggests caution regarding a putative association between sarcoidosis and *B. burgdorferi*.

What seems clear is that more than one infectious agent might be implicated in the pathogenesis of sarcoidosis; probably the patient’s geographical location might dictate which microorganisms are more involved.

More studies and clinical trials are needed to extend this evidence to a more global level.

## Additional files

Additional file 1: PRISMA Checklist (PDF 545 kb)
Additional file 2: Search strategy of MEDLINE via OVID. (DOC 22 kb)
Additional file 3: Flow diagram of the current meta-analysis. (DOC 1550 kb)

## Abbreviations

*B. burgdorferi*: Borrelia burgdorferi
CI: Confidence interval
HHV-8: Human herpesvirus 8
MTBC: Mycobacterium tuberculosis complex
NTM: Nontuberculous mycobacteria
OR: Odds ratio
*P. acnes*: Propionibacterium acnes
PRISMA: Preferred Reporting Items for Systematic Reviews and Meta-Analyses
