# Supplementary material for: Is there any association between Sarcoidosis and infectious agents?: a systematic review and meta-analysis
Source: BMC Pulm Med. 2016 Nov 28;16:165. doi: 10.1186/s12890-016-0332-z (PMC5126827; doi:10.1186/s12890-016-0332-z)
Supplement: Additional file 2: — Search strategy of MEDLINE via OVID. (DOC 22 kb) [file 12890_2016_332_MOESM2_ESM.doc]

**ADDITIONAL FILE 2**

**Search Strategy for MEDLINE via OVID**

**1** exp SARCOIDOSIS/

**2** sarcoid$.mp.

**3** 1 or 2

**4** exp INFECTIONS, BACTERIAL/

**5** exp BACTERIA/

**6** infectio$.mp.

**7** mycobacteri$.mp.

**8** propionibacterium.mp.

**9** exp DISEASES, VIRUS/

**10** exp VIRUSES/

**11** virus$.mp.

**12** herpesvirus.mp.

**13** exp MYCOSES/

**14** fungus.mp.

**15** exp DISEASES, PARASITIC/

**16** parasit$.mp.

**17** 4 or 5 or 6 or 7 or 8 or 9 or 10 or 11 or 12 or 13 or 14 or 15 or 16

**18** 3 and 17

+ RCT filter tested by Cochrane Collaboration
