# Supplementary figures and images for: Is there any association between Sarcoidosis and infectious agents?: a systematic review and meta-analysis
Source: BMC Pulm Med. 2016 Nov 28;16:165. doi: 10.1186/s12890-016-0332-z (PMC5126827; doi:10.1186/s12890-016-0332-z)

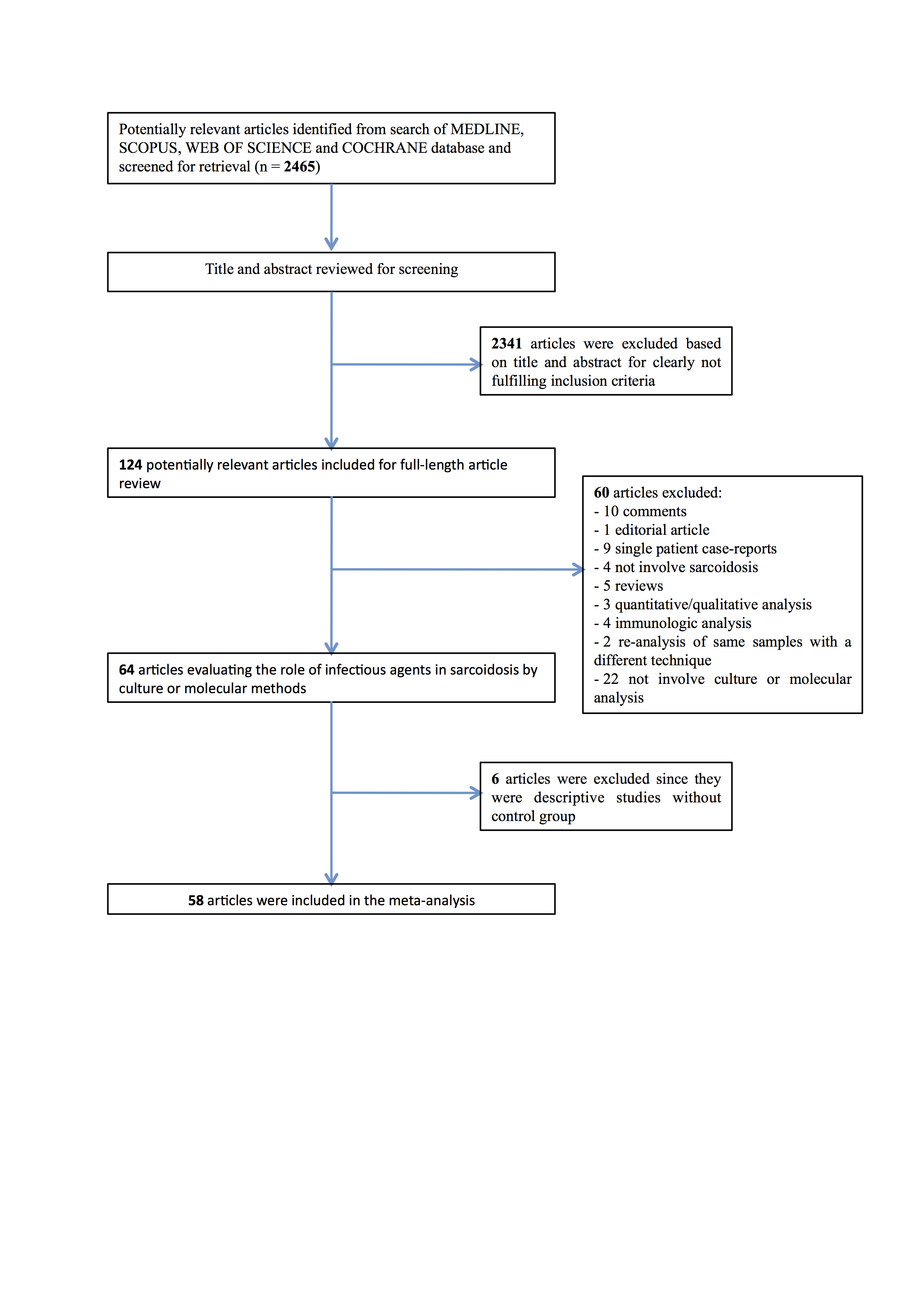


**ADDITIONAL FILE 3**

Supplement: Additional file 3: — Flow diagram of the current meta-analysis. (DOC 1550 kb) [file 12890_2016_332_MOESM3_ESM.doc]
